# Brain–body responses to chronic stress: a brief review

**DOI:** 10.12703/r/10-83

**Published:** 2021-12-16

**Authors:** Brandon L Roberts, Ilia N Karatsoreos

**Affiliations:** 1 Department of Psychological and Brain Sciences, Neuroscience and Behavior Program, University of Massachusetts Amherst, Amherst, MA, USA

**Keywords:** Allostatic Load, Burnout, Stress physiology, Neurobiology, Resilience, Vulnerability, Inflammation, Metabolism

## Abstract

In order to survive and thrive, organisms must adapt to constantly changing environmental pressures. When there are significant shifts in the environment, the brain and body engage a set of physiological and behavioral countermeasures collectively known as the “stress response”. These responses, which include changes at the cellular, systems, and organismal level, are geared toward protecting homeostasis and adapting physiological operating parameters so as to enable the organism to overcome short-term challenges. It is the shift of these well-organized acute responses to dysregulated chronic responses that leads to pathologies. In a sense, the protective measures become destructive, causing the myriad health problems that are associated with chronic stress. To further complicate the situation, these challenges need not be purely physical in nature. Indeed, psychosocial stressors such as ruminating about challenges at work, resource insecurity, and unstable social environments can engage the very same emergency threat systems and eventually lead to the same types of pathologies that sometimes are described as “burnout” in humans. This short review focuses on very recent empirical work exploring the effects of chronic stress on key brain circuits, metabolism and metabolic function, and immune function.

## Introduction

As a term, “stress” is loaded. It carries with it significant negative connotations and is commonly used in media and marketing as a “boogeyman” that needs to be banished from one’s life. However, this overly simplistic view completely misses the importance of stress and the stress response in the maintenance of health and survival when the environment puts significant pressure on the brain and body. An important distinction is the difference between *eustress* and *distress*. These terms are part of a broader theme that there exist instances where the stress responses that are engaged improve performance and outcomes (eustress) and situations where stressors lead to negative outcomes and pathophysiology (distress). It is therefore critical to understand what we mean by the negative aspects of stress and how we operationalize these concepts in our research and practice. This review attempts to clarify some of these concepts using recent literature in the realm of chronic stress and its effects on mental and physical well-being.

## Stress and the hypothalamic–pituitary–adrenal axis

Although a detailed review of the neural and neuroendocrine factors that govern the stress response exceeds the remit of this short review, it is important to understand the various players in the brain and periphery that transduce environmental challenges into a complex suite of behavioral and physiological countermeasures which enable an individual to cope^1–4^. The central mediator of these “alarm” or “emergency” responses is the hypothalamic–pituitary–adrenal (HPA) axis. It is important to understand that these circuits are activated in different ways under conditions of both psychological stress and more physiological stressors (for example, blood loss and immune challenge)^2,4,5^. Arguably, in many cases, it is difficult to fully tease apart psychological and physiological stress and this issue is certainly beyond the scope of this review. At the core of this response is the paraventricular nucleus (PVN) of the hypothalamus, which directly receives information about immediate risks to homeostasis via other neurons and brain regions that serve as first- or second-order relays^1,5^. This can include physiological states such as dehydration, starvation, blood loss (shock), and immunological challenges^5^. The PVN relays these signals through a combination of descending noradrenergic projections to the adrenal gland or via neuroendocrine responses through the release of corticotropin-releasing hormone into the median eminence, causing the release of adrenocorticotropic hormone (ACTH) from the anterior pituitary into the general circulation which upon arrival at the adrenal gland stimulates the release of corticosteroids (cortisol or corticosterone [CORT]) from the adrenal cortex^6^. These adrenal steroid hormones then drive many (though not all) of the changes that we will discuss in the rest of this review through both classic genomic steroid hormone effects and non-genomic effects (depending on the cell, tissue, and timing/duration of the exposure). It is important to understand that in addition to this classic emergency response system, the HPA axis has a wider set of brain inputs that provide information about anticipatory stressors (that is, stressors that undergo significant processing and classification before an appropriate HPA response is engaged)^1,2,5^. This process involves key brain-stem areas such as the nucleus of the solitary tract (NTS) (a key relay of all manner of information about peripheral states via the vagus nerve) as well as limbic regions such as the prefrontal cortex (PFC), amygdala (AMG), and hippocampus (HIPP)^1^. These regions form a web of interconnected stimulatory and inhibitory inputs to the HPA axis which, under normal circumstances, ensure that stress responses are engaged only under appropriate conditions^2,5^. This process of adaptive coordinated responding can be considered as engaging “allostatic” responses (that is, brain and body responses orchestrated to anticipate and counter environmental challenges to homeostasis) rather than simply responding to emergencies after they occur^4^. The rest of this review will focus on situations when these allostatic neural and neuroendocrine systems begin to be overused or malfunction, as is the case with chronic stress exposure. Repeated activation of these circuits can lead to an inappropriate engagement of the HPA axis and a dysregulation of the stress response causing under- or over-responding of the HPA or an inefficient termination of the HPA response (or both). These non-optimal responses can lead to “wear and tear” on the brain and body, conceptualized as “allostatic load” which can have cumulative long-term impacts on mental and physical health as they lead to pathological changes in the brain and periphery^3^.

## Chronic stress and the brain

It is important to note that acute and chronic stress impact nearly all aspects of neural and glial function but that this review is purposely limited in scope primarily to neurons in the limbic system. Neural circuits within the limbic system that regulate fear, executive function, learning, and memory are particularly sensitive to chronic stress, and recent findings emphasize important roles of the cell- and pathway-specific effects of stress. Several detailed reviews explore the state of this growing and complex field, highlighting links between the effects of stress on brain circuits in humans and non-human animals and how these relate to other neuropsychiatric outcomes like depression and anxiety^7,8^. Chronic stress alters dendritic density and complexity throughout the brain. Within the dentate gyrus of the HIPP, chronic stress has opposing effects on the dendritic complexity of granule cell layer (GCL) neurons, being dependent upon their projection site. Specifically, chronic stress leads to increased dendritic volume in neurons terminating in the inner medial layer while leading to both decreased dendritic volume and length in the more distant projecting medial and outer molecular layer neurons^9^. The mechanisms of these changes are likely myriad, but GABA signaling is suggested to promote dendritic complexity within the HIPP. In parallel with the anatomical findings, this paradigm of chronic stress also increased levels of glutamate and GABA within the GCL of the dentate gyrus^9^.

Chronic stress can induce neuronal remodeling within the AMG and is associated with increased anxiety and post-traumatic stress disorder (PTSD)-like behaviors^4^. At the circuit level, this occurs in a highly site-specific manner. In the basolateral AMG (BLA), chronic restraint stress (CRS) increases the number of mature dendritic spines and increases excitatory glutamatergic signaling, particularly in neurons projecting to the ventral HIPP, while having little effect on spine density or cell excitability in BLA neurons projecting to the dorsal medial PFC (dmPFC) or nucleus accumbens^10,11^. While the cellular mechanisms here are also likely multifaceted, recent work shows that CRS produces a calcium-activated potassium (SK2) channel-dependent activation of the BLA to ventral HIPP projecting neurons, specifically in the BLA neurons receiving inputs from the dmPFC^12^.

Although recent studies show little effect of CRS on BLA to dmPFC projecting neurons at the level of the AMG, CRS can alter presynaptic glutamate release from these neurons at the level of the PFC and this remarkably occurs in a mouse strain–specific manner. Of note, CRS leads to increased glutamate release in C57BL/6J mice and decreased release in DBA/2J mice and the latter strain also shows higher vulnerability to anxiety-like behaviors. Surprisingly, optogenetic photostimulation of these BLA to dmPFC neurons increased anxiety-like behaviors in C57BL/6J mice^10^. Adding more complexity to this picture is the fact that timing of chronic stress can also significantly impact these circuits, and early life stress is able to shift the excitatory–inhibitory balance of layer II/III neurons of the infralimbic cortex by decreasing the number of excitatory inputs^13^.

The AMG and PFC have reciprocal connections to each other, and CRS induces increased glutamatergic release from mono-directional medial PFC-to-BLA inputs, a circuit that regulates fear conditioning^14^. Repeating the common theme of regional and cell-type specificity, distinct neuronal populations in the PFC seem vulnerable to stress. Glutamatergic pyramidal neurons (PYR) in the medial PFC send projections throughout the brain, including the AMG, and can be divided into dopamine receptor 1 (D1)- and D2-containing PYR neurons, which have little overlap in expression^15^. Chronic unpredictable stress produces increased anxiety and depression-like behaviors, and within the prelimbic region of the PFC, promotes plasticity via increases in excitatory and inhibitory inputs onto D1 but not D2 PYR neurons^15^. Recent work has also demonstrated that stress can alter brain states more broadly, including changes in hippocampal synchrony^16^ and gamma oscillations in the nucleus accumbens^17^. Such states also potentially predict vulnerability to depression^18^, which we know is also significantly modified by chronic stress^7,8^. Taken together, these results further our understanding of the effects of chronic stress on the PFC, AMG, and HIPP as it relates to connectivity and informational throughput. Moreover, these results emphasize the importance of cell type and animal strain specificity in dissecting neural circuits impacted by stress – key details that are often overlooked.

## Chronic stress and metabolism

As noted in our introduction, stress systems are crucial for engaging optimal protective responses in the face of threat. A key aspect of physiology impacted at both the whole-organism and cellular levels is metabolism. For instance, a major role of glucocorticoids is regulating glucose release from multiple tissues into the bloodstream, thus increasing available energy^19^. Mobilizing energy stores is a necessary step in the face of environmental challenge, and rebuilding those stores following termination of stress is similarly necessary to ensure that an organism can face future threats effectively. This reality inextricably links stress and energy metabolism. Overuse of these pathways and inefficient regulation of metabolic systems during and following stress are thus potential major contributors to the pathology of chronic stress. As noted earlier, given the focused nature of this short review, readers are directed to multiple excellent reviews that provide an in-depth look into the history of links between acute and chronic stress and metabolic function^20–23^.

Environmental stress and stress hormones such as glucocorticoids are tightly coupled with behavioral and physiological components of energy homeostasis in humans and non-human animals. At the whole-organism level, environmental stress leads to reductions in impulse control, changes in food intake, and dysregulated metabolic states. At the cellular level, chronic stress alters cellular organization, mitochondrial function, glucose regulation, and signaling of inflammatory markers^23^. The impact of chronic stress on metabolic function is distinct from those of acute stress. Both result in acute anorexigenic effects; however, chronic stress can lead to persistent decreases in body weight independently of food intake. Recent evidence proposes that this increase in energy expenditure is due, in part, to an increase in brown adipose tissue and increased expression of the mitochondrial carrier protein uncoupling protein 1 (UCP1)^24^.

Chronic stress effects on energy homeostasis are widely attributed to increases in glucocorticoid levels through activation of the HPA axis^25^. Glucocorticoids play an integral and bi-directional role in neuropsychiatric and metabolic states, and disruption of this system, such as in Cushing syndrome, can result in hypertension, insulin resistance, adiposity, neuronal damage, depression, and anxiety^26,27^. Our group characterized a potential animal model for Cushing syndrome, showing that treatment with CORT in the drinking water of adult mice results in increased adiposity, decreased glucose tolerance, and elevated plasma insulin^28^. It is noteworthy that these effects occur independently of signaling of ghrelin receptor^29^, a key metabolic peptide. Surprisingly, developmental state also modulates this response, and adolescent mice that receive high CORT show decreases in bone density in addition to the metabolic outcomes demonstrated in adult mice^28^.

Although glucocorticoids are a link between chronic stress and metabolic dysfunction, recent work demonstrates that chronic stress may induce glucocorticoid-independent damage to neurons critical for sensing glucose. For example, CRS-induced hyperglycemia in mice produced apoptosis of glucose-sensitive neurons in the NTS, a termination site of gastric vagal afferents and a key region in cardiovascular and metabolic regulation^30^. However, mice that received the synthetic glucocorticoid dexamethasone (DEX) did not show similar changes. Although, it should be noted that DEX does not easily cross the blood–brain barrier without the P-glycoprotein transporter and thus a lack of central effects should always be taken into consideration with this in mind^31,32^. CRS also increased adrenal weight and adrenal cortex thickness, whereas DEX-treated mice displayed adrenal atrophy and decreased adrenal cortex thickness^30^. This further supports the notion that chronic stress engages a constellation of effects that can have significant negative outcomes which cannot be replicated by simply treating with glucocorticoids.

Although non-human animal models are critical for understanding mechanistic links between stress, glucocorticoids, and metabolic function, there is ample clinical and epidemiological work in humans demonstrating the significance of these interactions for health. Most recently, new work has established that obesity rates are higher in children from low-income households, and an increasing number of studies demonstrate that the chronic stress associated with food insecurity negatively impacts children’s emotional well-being^33^. In households with high parent-perceived stress, children have a higher body mass index and rates of consuming fast food compared with low parent-perceived stress households, particularly in households from marginalized ethnic groups^34^. Home food availability is often dependent on parental food preparation, and recent work shows a negative correlation between parental stress and the healthy food availability for children^35^. There has been much work exploring how acute stress affects food seeking and craving behaviors while effects of chronic stress are sparser. Notably, a recent controlled trial suggests that perceived stress does not increase the preference for highly palatable food in adults^36^.

While changes in classic aspects of metabolism (for example, adiposity, feeding hormones, and energy expenditure) are evident in chronic stress, there is also increasing evidence of the important links between gut microbiota and overall metabolic health. The composition of gut microflora is both affected by, and modulates resilience to, chronic stress. This likely occurs through effects on the immune system^37,38^. Supporting this bi-directional relationship, emerging data from human trials support the relationship between stress and gut health. The administration of the probiotic *Lactobacillus gasseri* CP2305 in young adults exposed to chronic stress and in a cohort of medical students decreased perceived stress, improved mental state, and attenuated stress-associated changes in gut bacterial flora^39,40^. Together, these findings highlight the complex relationship between stress, societal factors, and energy intake. 

## Chronic stress and inflammation

There are many links between stress and the immune system, including an emerging consensus that the gut microflora is affected by chronic stress and may contribute directly to stress resilience and vulnerability (as described above). For decades, links between stress and immune function have been well documented, and there is current agreement that stress has both positive and negative effects in this realm. On the one hand, stress can activate the immune system, shifting it into a more “alerted” state such that any infections might be quickly detected and isolated/irradicated^41^. In this way, under acute conditions, we can consider the stress and immune responses to be working toward similar goals: short-term allostatic responses that should lead to a rapid conclusion of the stress-inducing situation and its immediate aftermath. However, chronic physical and psychosocial stress leads to a significant perturbation of immune responses and can lead to hyper-inflammatory responses, many of which can resemble chronic inflammatory diseases^42^ and can even impact cancer progression^43–45^. Exciting recent work further demonstrates that chronic stress can result in low brain vasculature and elevated permeability of the blood–brain barrier^46–48^, potentially leading to vulnerability in penetration of peripheral immune cell types. Specific effects of chronic stress on the response of several classes of immune cells, including on regulatory T cells, which can protect from adverse stress-induced behaviors when balanced by healthy metabolites of the gut microbiota, have also recently been reported^49^. These findings provide several clear pathways by which chronic stress can impact central and peripheral inflammatory processes.

Exciting work over the past decade has implicated microglia, the primary immunosurveillance cells of the brain, as causal agents in many neurobehavioral changes following chronic stress^50^. For instance, increases in microglia (particularly in the activated state) are reported in many of the brain areas that are significantly impacted by chronic stress, including the PFC and HIPP, although timing and sex modulate these responses^51–53^. Notably, recent work has demonstrated that exposure to chronic stress during adolescence (noted earlier in this review as a stress-sensitive period of exposure to glucocorticoids) can lead to significant changes in the neuroimmune transcriptome, likely marking a significant shift of developmental trajectories^54^. The role of the immune system as both a responder and modulator of the stress response is a major area of new research and certainly represents another important nexus between brain and periphery in the response to chronic stress.

## COVID-19 pandemic as chronic stress: early insights

The recent (and continuing) COVID-19 global pandemic has resulted in a unique set of circumstances that has disrupted daily life and produced significant chronic psychosocial and physical stress, including increases in social isolation, financial and job insecurity, and sedentary behavior and a decrease in those seeking necessary medical care^55–57^. These indirect consequences of the pandemic have highlighted the relationship between eating patterns, physical activity, sleep–wake cycles, and stress-related mental illness. For example, concomitant with declines in physical activity, the pandemic led to increases in weight gain and anxiety scores, both of which had a larger-magnitude impact on people with obesity^56^. As noted earlier, obesity levels are higher in children from low-income households and food insecurity stress negatively impacts emotional well-being^33^. Given the increased emotional vulnerability of early adolescents and the long-term consequences associated with childhood obesity, the increases of emotional distress and altered lifestyle choices inflicted by the COVID-19 pandemic will require a range of longitudinal studies before we can fully comprehend the long-term health consequences associated with the pandemic^58^. Already, there is growing consideration of the impact of the pandemic on chronic stress and burnout in physicians^59^ as well as the critical question of how the poorer COVID-19 outcomes in different racial and ethnic groups might be related to underlying inflammatory states driven by the chronic psychosocial and environmental stress experienced by these individuals^60^.

## Summary/Conclusion

Stress is a multifaceted concept that too often is associated with negative connotations. In the acute sense, stress encompasses necessary, adaptive, and even helpful responses to homeostatic threats. The negative connotations of the term stress are likely earned when considering the effects of *chronic* stress or *dis*tress. In these instances, the repeated activation of stress pathways and engagement of allostatic mediators can lead to allostatic overload and long-term damage to the same systems that are critical for protecting the organism. In a sense, chronic stress can be conceptualized as the set of events that leads normally *con*structive responses to become *de*structive, leading to pathological outcomes in both central and peripheral systems, perhaps best described in the clinical literature (and common parlance) as “burnout”^61^. We have limited our present discussion to a subset of neural circuits, a handful of components of the metabolic system, and a small portion of the immune system which hopefully highlights some recent findings that illustrate this key concept. These short descriptions surely leave out many more findings over the past decade that are beyond the scope of this review but further emphasize the importance of approaching the causes and consequences of chronic stress from a brain–body interaction perspective and not purely a brain or periphery viewpoint. Future work should be aimed at understanding the mechanisms at the cellular, organismal, and even societal levels that help to buffer stress responses and prevent the transition to these destructive responses. In addition, understanding the factors that modulate an individuals’ resilience or susceptibility to this transition, and the factors that mediate the destructive pathological aspects of chronic stress, should be of central importance for new studies^3^. An area of growing interest is understanding how the circadian (daily) clock and sleep impact stress responses, particularly in the context of our modern 24-hour “always on the go” society. Several recent reviews clearly demonstrate that disrupted light–dark cycles, inappropriate exposure to light at night, and sleep disruptions all may contribute to the buildup of allostatic load, potentially by impairing key physiological systems from operating optimally with each other^3,62^. By gaining appreciation of the factors that help to mitigate the negative outcomes of stress as well as those that promote pathologies, we might be better able to devise treatment strategies or even prophylactic approaches to halt, or hopefully reverse, the negative consequences of chronic stress exposure.
